# Camel Milk Alleviates Chronic Fatigue Syndrome-like Symptoms in Mice by Modulating the Small Intestinal Microbiota and Inflammation

**DOI:** 10.3390/foods15142451

**Published:** 2026-07-10

**Authors:** Shiqi Zhang, Yating Wu, Fulan Wang, Hongman Li, Nan Zheng, He Chen, Yankun Zhao

**Affiliations:** 1 Laboratory of Quality and Safety Risk Assessment for Agro-Products of Ministry of Agriculture and Rural Affairs, Key Laboratory of Agro-Products Quality and Safety of Xinjiang, Urumqi 830091, China; 2 Institute of Quality Standards & Testing Technology for Agro-Products, Xinjiang Academy of Agricultural Sciences, Urumqi 830091, China; 3 State Key Laboratory of Animal Nutrition and Feeding, Institute of Animal Sciences, Chinese Academy of Agricultural Sciences, Beijing 100193, China

**Keywords:** chronic fatigue syndrome, camel milk, gut microbiota, intestinal barrier, microbiota–gut–brain axis, systemic inflammation

## Abstract

This study aimed to investigate the therapeutic effects of camel milk (CM) on chronic fatigue syndrome (CFS) and elucidate the mechanisms underlying the microbiota–gut–brain axis. Using a murine model of CFS induced by chronic restraint and forced swimming stress, we administered lyophilized CM (1500 mg/kg/day, equivalent to approximately 121.5 mg/kg/day in humans based on body surface area conversion using the standard allometric scaling formula) orally. CM supplementation was significantly associated with ameliorated fatigue-like behaviors, as evidenced by prolonged swimming endurance and reduced immobility time. Metagenomic analysis revealed that CM was associated with reshaping of the small intestinal microbiota, including enrichment of beneficial *Lactococcus lactis* and suppression of pathobionts (*H. hepaticus* and *H. typhlonius*). These microbial shifts correlated with increased luminal lactic acid, improved intestinal barrier integrity (increased villus height, reduced crypt depth), and attenuated local inflammation (reduced TNF-α and IL-6, elevated IL-10). Consequently, CM was associated with reduced bacterial translocation and systemic inflammation, and normalization of hypothalamic–pituitary–adrenal (HPA) axis hyperactivity. We conclude that CM is associated with prevention of CFS-like symptoms through modulation of the gut ecosystem and strengthening of the intestinal barrier, potentially breaking the vicious cycle of gut inflammation and HPA axis dysfunction, although causality remains to be established through fecal microbiota transplantation or similar mechanistic studies.

## 1. Introduction

Chronic fatigue syndrome (CFS), also termed myalgic encephalomyelitis (ME), is a multifaceted condition characterized by enduring, debilitating fatigue that persists despite rest, frequently co-occurring with cognitive disturbances, sleep abnormalities, and myalgia [1]. Globally, the prevalence of CFS is estimated at ~0.89%; however, epidemiological data from China indicate a markedly higher rate of 12.54%. The condition predominantly affects individuals aged 31–50 years, with a notable female predominance [2]. This increase may be associated with modern lifestyle factors, including high work-related stress and sedentary behavior, particularly among urban professionals. More concerningly, CFS is increasingly recognized as a risk factor for severe health events and may contribute to a notable proportion of work-related sudden deaths in China, rendering it a serious public health issue.

The exact cause of CFS is still unknown, but it likely stems from a mix of genetic susceptibility [3], stress [4], immune problems [5], metabolic issues [6], and viral infections [7]. Recently, attention has focused on the gut–brain axis. In many CFS patients, the gut microbiome appears dysbalanced, characterized by lower diversity and higher abundance of harmful bacteria. This disrupts the production of short-chain fatty acids (SCFAs) and weakens the intestinal barrier [8,9]. As a result, the gut lining becomes eroded, tight junction proteins are lost, and lipopolysaccharide (LPS) leaks from the gut into the bloodstream [10]. This “leaky gut” triggers widespread inflammation, raising levels of cytokines like IL-6 and TNF-α, and increases oxidative stress [11]. Such inflammation can interfere with energy production—specifically mitochondrial ATP synthesis—and alter neurotransmitter pathways, such as the tryptophan–5-HT axis [12]. Over time, these changes affect the nervous system, leading to vagal nerve dysfunction, microglial activation, and issues with the HPA axis and cognition [13]. This model is supported by findings that CFS severity links to serum LPS antibodies [14] and that metabolites like butyrate can reduce neuroinflammation [15].

Because gut inflammation and dysbiosis seem central to CFS, treatments targeting the gut are worth exploring [16]. Researchers are focusing increasingly on non-pharmacological approaches, particularly dietary interventions that may help restore balance to the gut–brain axis [17]. Camel milk (CM) is one such dietary candidate that has drawn attention. It is rich in proteins, fatty acids, vitamins, and bioactive compounds like immunoglobulins and lactoferrin, which have shown antioxidant and anti-inflammatory effects in previous studies [18]. Some research suggests CM can shift the gut microbiome by increasing beneficial bacteria and reducing harmful ones, which may boost short-chain fatty acid levels and strengthen the gut barrier [19,20]. Components like lactoferrin might also directly help tighten intestinal junctions to stop LPS leakage [21], and the tryptophan in CM could support serotonin production, potentially aiding mood and cognition [22]. Yet, exactly how CM influences microbial structure to reduce small intestinal inflammation and relieve CFS symptoms is not fully understood.

More recently, Ali et al. [21] conducted a comprehensive systematic review on camel milk digestibility and its effects on gut microbiota, concluding that camel milk proteins are more readily digestible than bovine milk proteins due to the absence of β-lactoglobulin and the presence of smaller casein micelles, which may enhance the bioavailability of bioactive peptides in the distal intestine. Notably, camel milk is uniquely rich in immunoglobulins (predominantly IgG) and lactoferrin, which exhibit remarkable stability and bioactivity under gastrointestinal conditions. Freeze-drying processing, as employed in the present study, preserves the structural integrity and bioactivity of camel milk immunoproteins more effectively than thermal processing, maintaining their capacity to bind bacterial lipopolysaccharides and modulate epithelial inflammatory responses. Mechanistically, camel lactoferrin exerts gut-protective effects through multiple pathways: (i) direct iron chelation that limits pathogen growth, (ii) binding to Toll-like receptor 4 (TLR4) on intestinal epithelial cells to competitively inhibit LPS-induced NF-κB activation, and (iii) upregulation of tight junction proteins (occludin and claudin-1) via activation of the PI3K/Akt signaling pathway. Camel immunoglobulins, particularly secretory IgA, contribute to gut barrier function by forming a protective immune exclusion layer on the mucosal surface, neutralizing luminal pathogens, and preventing bacterial translocation. These bioactive components together create an immuno-protective environment that synergizes with microbiota-modulating effects to strengthen intestinal barrier integrity [23].

In this study, we investigated the mechanism of camel milk using a chronic fatigue syndrome mouse model, focusing on the microbiota–gut–brain axis. We hypothesize that camel milk prevents fatigue by reshaping the gut microbiome (particularly reducing harmful bacteria), thereby alleviating intestinal damage and inflammation, and ultimately breaking the vicious cycle of systemic inflammation. By linking changes in microbial composition to gut health, inflammatory markers, and behavioral outcomes, we aim to elucidate whether camel milk can serve as a practical dietary intervention for treating chronic fatigue syndrome.

## 2. Methods

### 2.1. Camel Milk Collection and Preparation of Lyophilized Powder

Raw milk was collected from 89 healthy lactating Bactrian camels (Camelus bactrianus) at mid-lactation (approximately 3–6 months postpartum) at Dabancheng Dairy Station, Xinjiang, China. Daily milk samples from all camels were pooled immediately after milking and thoroughly homogenized to generate a single bulk sample (approximately 300 mL). This process was repeated on six consecutive days, yielding six independent biological replicates. Pooled samples were aseptically transferred to pre-chilled amber glass bottles, snap-frozen on dry ice, and stored at −80 °C. Lyophilized camel milk powder was prepared by freeze-drying thawed samples (FreeZone^®^ 2.5 L Benchtop Freeze Dryer, Labconco, Kansas City, MO, USA; −50 °C, 10 Pa, 48 h). The nutritional composition and microbiological quality of the lyophilized camel milk powder were determined using standard analytical methods. Protein content was measured by the Kjeldahl method (N × 6.38), fat content by the Gerber method, and lactose content by an enzymatic method. Total viable bacterial count was assessed by standard plate count according to the Chinese national standard method (GB 4789.2 [24]). The results showed that the lyophilized camel milk powder contained 26.4% protein, 28.7 ± 0.38% fat, and 35.2 ± 0.52% lactose, with a total bacterial count of less than 1.0 × 10^3^ CFU/g, indicating satisfactory microbiological quality for oral administration in animal experiments. All analyses were performed in triplicate, and results are expressed as mean values.

### 2.2. Animal Housing and Acclimatization

Male BALB/c mice (6 weeks old, 18–22 g; SPF Biotech, Suzhou, China; SCXK [Jiangsu] 2022-0006) were acclimatized for 7 days under standard conditions (22 ± 2 °C, 50 ± 10% humidity, 12 h light/dark cycle) with free access to standard rodent diet (GB 14924.3-2010 [25]) and purified water.

### 2.3. Preparation of Test Materials

Lyophilized camel milk powder was produced as described in Section 2.1. For administration, the powder was freshly reconstituted in distilled water. Rhodiola capsule (Weihai ZiGuang Bio-Tech Development Co., Ltd., Weihai, China; batch No. A0524031), a commercially available dietary supplement, was used as the positive control. The capsule contains Rhodiola rosea extract standardized to 3% salidroside and 1% tyrosol, which are known for their adaptogenic, anti-fatigue, and anti-inflammatory properties, and was similarly dissolved in distilled water.

### 2.4. Experimental Design and Dosing

Forty-two male BALB/c mice were randomly assigned to six groups (n = 7 per group) using a computer-generated random number sequence: (1) control; (2) model (cold-water swimming + chronic restraint); (3) rhodiola capsule (600 mg/kg/day BW, Weihai ZiGuang Bio-Tech; A0524031); (4)–(6) lyophilized camel milk powder (500, 1000, 1500 mg/kg/day BW). CFS-like conditions were induced in all groups except the control using a dual-stress paradigm over 56 consecutive days. Specifically, mice were subjected to daily physical restriction within well-ventilated tubes (3 h per session), immediately followed by a 20 min swimming bout in temperature-controlled water maintained at 22 °C. This protocol, which integrates psychological and physical stressors, was adopted from established methodologies [26,27]. The first two weeks served as modelling adaptation periods, with dietary intervention commencing from the third week. Test materials were administered via oral gavage (10 mL/kg) daily. Body weight and food intake were monitored weekly. Sample size (n = 7 per group) was determined based on pilot data and previous studies [23,26], assuming a 30% difference in forced swimming time, a standard deviation of 20%, 80% power, and α = 0.05. The three doses (500, 1000, and 1500 mg/kg/day) were selected based on the following considerations: (i) the low dose (500 mg/kg/day) corresponds to the lower range of camel milk consumption reported in traditional use and preliminary mouse studies [22]; (ii) the medium dose (1000 mg/kg/day) reflects the average consumption reported in human observational studies, approximating 250 mL of raw camel milk daily based on allometric scaling; and (iii) the high dose (1500 mg/kg/day) was chosen to explore potential dose-dependent effects and ensure the detection of therapeutic efficacy, given the relatively short intervention period (8 weeks) and potential variability in bioactive compound bioavailability. Notably, based on standard allometric scaling [28], the high dose corresponds to a human equivalent dose (HED) of approximately 121.5 mg/kg/day, which falls within the typical consumption range (250–500 mL/day) reported in human studies [23].

All animal procedures were performed with efforts to minimize pain and distress. All groups received the same standard rodent diet (GB 14924.3-2010 [25]) ad libitum throughout the study; the only difference between groups was the oral gavage supplement administered daily. During the 56-day chronic restraint and forced swimming protocol, mice were monitored twice daily (8:00 AM and 8:00 PM) for signs of distress, including piloerection, hunched posture, lethargy, and reduced responsiveness. For any potentially painful manipulation (e.g., oral gavage, blood collection), isoflurane anesthesia was used as described in Section 2.6. No unexpected adverse events or mortality occurred in any group throughout the study. Minor body weight loss was observed in the model group as part of the CFS phenotype (reported in Section 3.1), which was anticipated and was not considered an adverse event requiring intervention. Humane endpoints were predefined as: (1) loss of >20% of initial body weight within 72 h, (2) inability to reach food or water, (3) severe lethargy unresponsive to gentle stimulation, or (4) persistent signs of pain (e.g., vocalization, self-mutilation). None of the animals reached these endpoints, and therefore no early euthanasia was required.

### 2.5. Behavioral Assessment

Following the 8-week stress induction period, three behavioral assays were performed to evaluate locomotor function and fatigue-like behaviors: (i) an open field test to monitor spontaneous exploration over 5 min using EthoVision XT, Noldus Information Technology, Wageningen, The Netherlands; (ii) a tail suspension test quantifying immobility duration during the final 4 min; and (iii) a weight-loaded forced swim test measuring time to exhaustion under a 6% body-weight load.

### 2.6. Sample Collection and Processing

Following isoflurane anesthesia, blood was obtained through cardiac puncture using heparinized tubes. Animals were subsequently euthanized by cervical dislocation, and tissues including brain, small intestine, and luminal contents were rapidly harvested and cryopreserved in liquid nitrogen. Plasma was separated by centrifugation at 3000× *g* for 15 min at 4 °C, aliquoted, and stored at −80 °C until analysis. Brain tissues were weighed on ice and homogenized in ice-cold phosphate-buffered saline (PBS, pH 7.4) containing a protease inhibitor cocktail. The homogenates were centrifuged at 12,000× *g* for 20 min at 4 °C, and the supernatants were collected and stored at −80 °C for further analysis. Small intestinal contents (≈100 mg) were homogenized in ice-cold 80% methanol, centrifuged (14,000× *g*, 15 min, 4 °C), and supernatants used for metabolomic analyses.

### 2.7. Metagenomic Analysis of Microbial Composition and Function

Shotgun metagenomic sequencing was performed on small intestinal contents from three groups of mice: control (healthy), model (chronic fatigue syndrome-induced), and model + CM (model + 1500 mg/kg camel milk intervention, i.e., the high-dose group). The low- and medium-dose groups were not subjected to metagenomic or metabolomic profiling, as the primary objective of these analyses was to identify microbiota and metabolic signatures associated with the maximal therapeutic response (high-dose effect). Consequently, our mechanistic findings do not establish a dose-dependent relationship between microbiota changes and behavioral improvements; rather, they describe the microbial and metabolic state associated with the effective high dose. Future studies should include all dose groups in multi-omics analyses to elucidate dose-dependent microbial and metabolic mechanisms.

Total genomic DNA was extracted from the samples and used to construct paired-end libraries with an insert size of approximately 200–400 bp, following the manufacturer’s protocol for the DNBSEQ sequencing platform. The constructed libraries were sequenced on a DNBSEQ platform (MGI Tech Co., Ltd., Shenzhen, China) using a PE100 (paired-end 100 bp) strategy. The raw sequencing data were processed to generate high-quality clean reads by removing reads containing adapter sequences, reads with more than 10% of uncertain bases (N), and reads where over 50% of the bases had a Phred quality score below 20, using the SOAPnuke software (v1.5.6) [29].

To minimize potential interference from host DNA, the clean reads were aligned to the mouse reference genome (GRCm38/mm10) using Bowtie2 (v2.2.5) [30]. De novo assembly of the high-quality, host-depleted reads was performed for each sample individually using MEGAHIT (v1.0) with default parameters. Assembled contigs shorter than 300 bp were filtered out. Open reading frames (ORFs) were predicted from the assembled contigs using MetaGeneMark (v2.10). To construct a non-redundant gene catalog, all predicted genes were clustered using CD-HIT (v4.6.1) with a sequence identity threshold of 95% and a coverage threshold of 90% [31].

The abundance of each non-redundant gene in every sample was quantified using Salmon (v0.9.1) in mapping-based mode, and the results were normalized to transcripts per million (TPM) [32]. For functional annotation, the representative gene sequences from the non-redundant catalog were aligned against multiple public databases, including the Kyoto Encyclopedia of Genes and Genomes (KEGG), Clusters of Orthologous Groups (COG), and the Comprehensive Antibiotic Resistance Database (CARD), using the DIAMOND (v0.9.14) BLASTP algorithm with an e-value cutoff of 1 × 10^−5^ [33,34].

For taxonomic profiling, the high-quality, host-depleted reads were classified using Kraken2 (v2.0.7-beta) against a custom database comprising the NCBI Nucleotide (NT) database (202011 release) and the Unified Human Gastrointestinal Genome (UHGG) catalog [35,36]. The relative abundance of taxa at various levels (e.g., phylum, genus) was then estimated using Bracken2 (v2.0) [37].

### 2.8. Metabolomic Profiling and Analysis

Metabolomic profiling of small intestinal contents was performed using ultra-high-performance liquid chromatography coupled with quadrupole time-of-flight mass spectrometry (UHPLC-QTOF MS, Agilent 1290-6545, Agilent Technologies, Santa Clara, CA, USA). Chromatographic separation was achieved on a Waters HSS T3 column (2.1 × 100 mm, 1.8 μm; Waters Corp., Milford, MA, USA) maintained at 40 °C. The mobile phase consisted of (A) 0.1% formic acid in water and (B) 0.1% formic acid in acetonitrile, with a gradient elution program as follows: 0–2 min, 5% B; 2–6 min, 5% to 95% B; 6–8 min, 95% B; 8–8.5 min, 95% to 5% B; 8.5–10 min, 5% B (post-run equilibration). The flow rate was 0.3 mL/min, and the injection volume was 5 μL. The column temperature was maintained at 40 °C, and samples were kept at 4 °C in the autosampler throughout the analysis.

Mass spectrometry was performed in both positive and negative electrospray ionization (ESI) modes over a full-scan mass range of m/z 50–1500. The MS parameters were as follows: capillary voltage, 3.5 kV (positive) and 3.0 kV (negative); fragmentor voltage, 120 V; skimmer voltage, 65 V; drying gas temperature, 350 °C; drying gas flow, 10 L/min; sheath gas temperature, 400 °C; sheath gas flow, 11 L/min; nebulizer pressure, 35 psig. Data-dependent MS/MS acquisition was performed at collision energies of 10, 20, and 40 eV for structural confirmation of putative metabolites. Pooled quality control (QC) samples were prepared by combining equal aliquots (10 μL) from all individual samples and were injected every 10 analytical runs to monitor instrument performance, signal drift, and chromatographic reproducibility. Blank samples (solvent only) were injected at the beginning and end of each sequence to assess system contamination and carryover.

Data preprocessing, including feature detection, retention time alignment, and peak integration, was performed using the XCMS package (v3.14.0) in R (v4.3.1). Metabolite identification was performed at Level 2 (putatively annotated compounds) according to the Metabolomics Standards Initiative (MSI) guidelines, based on accurate mass matching (mass tolerance ≤ 5 ppm) and MS/MS spectral matching against the following databases: METLIN (https://metlin.scripps.edu, accessed on 22 December 2025), HMDB (v5.0, https://hmdb.ca, accessed on 23 December 2025), and MassBank (https://massbank.eu, accessed on 27 December 2025). Authentic standards were used for confirmation of key metabolites (lactic acid, 1-methylhistamine, indole-3-methyl acetate, and 8-oxo-7,8-dihydrodeoxyguanosine; Level 1 identification). For semi-quantitative analysis, peak areas of identified metabolites were normalized to the total ion count (TIC) of each sample to correct for run-to-run variations. No internal standard was used for absolute quantification; therefore, results are reported as relative abundance (normalized peak area) rather than absolute concentrations. Batch correction was not required as all samples were analyzed in a single analytical batch with QC samples interspersed throughout. The QC samples demonstrated a coefficient of variation (CV) of <15% for 90% of all detected features, confirming satisfactory analytical reproducibility. Data were log-transformed and Pareto-scaled prior to multivariate analysis.

Multivariate statistical analyses, including principal component analysis (PCA) and orthogonal partial least squares discriminant analysis (OPLS-DA), were performed using SIMCA v15.0 (Umetrics, Umeå, Sweden). Metabolites with variable importance in projection (VIP) ≥ 2.5 and unpaired *t*-test *p* < 0.05 were considered significantly different between groups. Pathway enrichment analysis was performed using MetaboAnalyst 6.0 (https://www.metaboanalyst.ca) with the mouse (Mus musculus) pathway library and the hypergeometric test for over-representation analysis. For differential metabolite identification, the initial screening criterion (VIP ≥ 2.5 and unpaired *t*-test *p* < 0.05) was used for feature selection. To control the false discovery rate (FDR) associated with multiple comparisons, the Benjamini–Hochberg FDR correction was subsequently applied to the selected metabolites, with an adjusted q-value threshold of < 0.05 considered statistically significant for the final metabolite set.

### 2.9. Hematoxylin and Eosin (H&E) Staining

A segment of the mid-jejunum (approximately 1 cm) was fixed in 4% paraformaldehyde for 24 h, dehydrated through a graded ethanol series, cleared in xylene, and embedded in paraffin. Serial sections (5 μm thick) were cut and stained with H&E for morphological evaluation. Villus height, crypt depth, and crypt density were quantified from H&E-stained slides using ImageJ software (v1.53k, NIH). Villus height was measured from the tip of the villus to the villus-crypt junction; crypt depth was defined as the distance from this junction to the base of the crypt. Crypt density was calculated as the number of crypts per millimeter of intestinal length across at least five well-oriented longitudinal sections per animal. All measurements were performed in a blinded manner by two independent observers.

### 2.10. Biochemical Analyses

Plasma levels of urea, lactate dehydrogenase (LDH), and lactate were measured using commercial kits (Nanjing Jiancheng Bioengineering Institute, Nanjing, China) according to the manufacturers’ instructions. Plasma concentrations of adrenocorticotropic hormone (ACTH), corticotropin-releasing hormone (CRH), and corticosterone were quantified using commercially available enzyme-linked immunosorbent assay (ELISA) kits, following the manufacturer’s instructions.

The concentrations of tumor necrosis factor-alpha (TNF-α), interleukin-6 (IL-6), interleukin-17 (IL-17), interleukin-17A (IL-17A), interleukin-1 beta (IL-1β), interleukin-10 (IL-10), and interferon-gamma (IFN-γ) in plasma and brain tissue supernatants were quantified using commercial ELISA kits (R&D Systems, Minneapolis, MN, USA) according to the manufacturer’s instructions. All samples were measured in duplicate. The optical density was read at 450 nm using a microplate reader. Cytokine concentrations were calculated from standard curves and expressed as pg/mL for plasma samples or as pg per mg of protein (pg/mg protein) for brain tissue samples. Total protein concentration in brain homogenates was determined using the bicinchoninic acid assay.

### 2.11. Statistical Analysis

All 42 mice completed the full 56-day experimental protocol; no animals or data points were excluded from subsequent analyses. Statistical analyses were performed using SAS version 9.4 (SAS Institute, Cary, NC, USA). One-way analysis of variance (ANOVA) was conducted using the PROC ANOVA procedure to compare group differences, followed by Tukey’s honestly significant difference test for post hoc multiple comparisons when a significant main effect was detected. Data were expressed as mean ± standard error of the mean (SEM). A *p* < 0.05 was considered statistically significant.

All outcome assessments (behavioral tests, biochemical assays, histomorphometry, and metagenomic analyses) were performed by investigators blinded to group allocation. Data were unblinded only after final statistical analysis. The randomization sequence was concealed from the experimenter until the end of the study. For parametric data, one-way ANOVA followed by Tukey’s HSD was used. For non-parametric data, Kruskal–Wallis test with Dunn’s post hoc was applied. For metagenomic differential abundance analysis, DESeq2 (v1.38.3) was used with the Benjamini–Hochberg false discovery rate (FDR) correction applied, and features with |log2FC| > 1 and FDR-adjusted q < 0.05 were considered significantly different between groups. STAMP analysis (v2.1.3) was also used for confirmation of differential taxa, with Welch’s *t*-test (for two-group comparisons) or one-way ANOVA (for multi-group comparisons) and the Benjamini–Hochberg FDR correction applied. All reported *p*-values are FDR-adjusted unless explicitly noted.

## 3. Results

### 3.1. Behavioral Performance and Plasma Fatigue Markers

**Figure 1:**
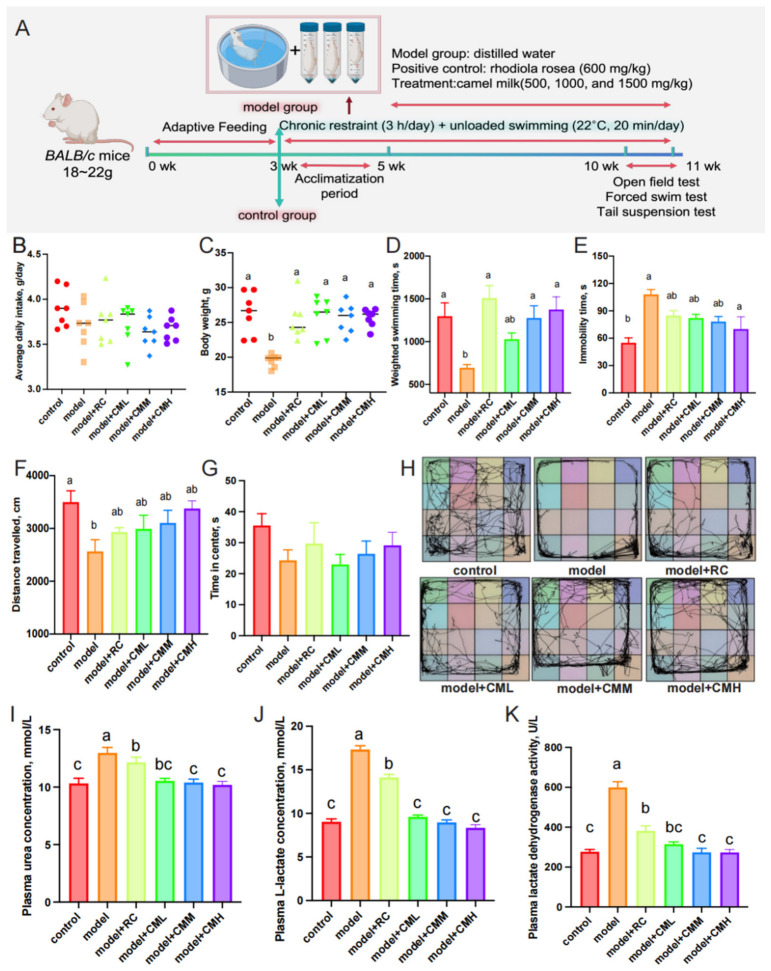
Behavioral performance and plasma metabolic biomarkers. (**A**) Schematic illustration of the experimental design. (**B**,**C**) Average daily food intake (**B**) and body weight changes (**C**) during the intervention period. (**D**,**E**) Exhaustion time in the weight-loaded forced swim test (**D**) and immobility time in the tail suspension test (**E**). (**F**–**H**) Open field test results: total distance traveled (**F**), time spent in the central zone (**G**), and representative heatmaps of locomotor trajectories (**H**). (**I**–**K**) Plasma biochemical markers: urea (**I**), lactate (**J**), and lactate dehydrogenase activity (**K**). Data are presented as mean ± standard error of the mean (SEM). Different letters (a, b, c) indicate statistically significant differences between groups (*p* < 0.05).

During the 56-day intervention period, mice subjected to chronic restraint and forced swimming stress exhibited features consistent with chronic fatigue syndrome, including reduced food intake and impaired weight gain (Figure 1A). The model group showed a significant reduction in average daily feed intake compared to the control group (*p* < 0.05; Figure 1B), while administration of lyophilized camel milk powder at medium (1000 mg/kg; CMM) and high (1500 mg/kg; CMH) doses significantly increased food intake (*p* < 0.05 vs. model). Correspondingly, body weight gain was markedly suppressed in the model group (Figure 1C), whereas both CMM and CMH groups exhibited significantly higher body weights than the model group (*p* < 0.05 vs. model).

In the weight-loaded forced swim test, swimming time was significantly shorter in the model group than in the control group (*p* < 0.05; Figure 1D), whereas the CMM and CMH groups showed prolonged endurance, with the CMH group exhibiting the most pronounced effect (*p* < 0.05 vs. model). In the tail suspension test, immobility time was significantly increased in the model group (*p* < 0.05; Figure 1E), and was significantly reduced in the CMM, CMH, and positive control (Rhodiola capsule) groups (*p* < 0.05 vs. model).

Open field testing revealed that the model group traveled a significantly shorter total distance (*p* < 0.05; Figure 1F) and spent less time in the center zone (*p* < 0.05; Figure 1G), indicating reduced spontaneous activity and increased anxiety-like behavior. Both CMM and CMH groups showed significantly greater locomotor activity and central exploration than the model group (*p* < 0.05 vs. model). Trajectory heatmaps (Figure 1H) visually confirmed these findings: the model group displayed restricted movement confined to the periphery, whereas treated groups exhibited broader, more dispersed patterns resembling those of controls.

Plasma biomarkers indicated metabolic perturbations in the model group: urea concentration (Figure 1I), lactate concentration (Figure 1J), and lactate dehydrogenase activity (Figure 1K) were all significantly elevated (*p* < 0.05). These levels were significantly reduced in the CMM and CMH groups (*p* < 0.05 vs. model). The positive control (Rhodiola capsule) group also showed significant improvements in behavioral parameters compared to the model group, with effects comparable to those observed in the CMH group, confirming the efficacy of the positive control and validating the experimental system. However, as the Rhodiola group was included primarily for behavioral validation, it was not subjected to the multi-omics profiling (metagenomic and metabolomic analyses) performed for the control, model, and model + CM groups.

### 3.2. Small Intestinal Microbial Community Structure

All samples yielded high-depth, high-quality metagenomic sequencing data. After stringent quality control (SOAPnuke), each sample retained an average of 200,361,270 clean reads (~20.0 Gb), with a clean data rate of 86.25%, confirming high library quality and suitability for reliable downstream analyses (Supplementary Table S1).

**Figure 2:**
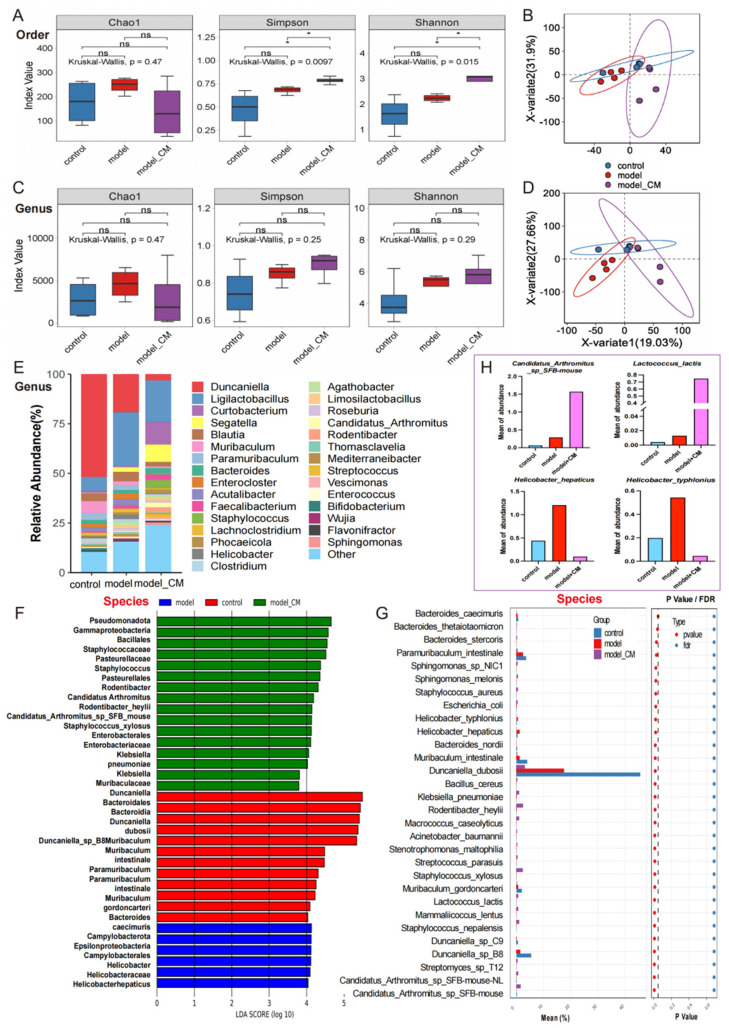
Microbiota composition and diversity. model _CM = model + CMH, 1500 mg/kg group. (**A**) Alpha diversity indices (Chao1, Simpson, and Shannon) at the order level. Statistical differences were assessed by Kruskal -Wallis test with Dunn’s post hoc comparisons (* = *p* < 0.05; ns = not significant). (**B**) Principal coordinate analysis (PCoA) based on Bray–Curtis dissimilarity at the order level; ellipses represent 95% confidence regions. (**C**) Alpha diversity indices at the genus level. (**D**) PCoA at the genus level; percentages indicate the proportion of total variance explained by each axis. (**E**) Relative abundance of the top 20 most abundant genera (>1% in at least one group); all remaining genera are aggregated as ‘other’. Taxa with significant differential abundance are further analyzed in panels (**F**–**H**). (**F**) LEfSe analysis of differentially abundant taxa (LDA score > 3.5); (**G**) STAMP analysis of differential microbial species (Welch’s *t*-test, FDR-adjusted q < 0.05); (**H**) Mean relative abundance of selected key species.

At the order level, the Shannon index was significantly higher in the model + CM group than in the control or model group (*p* = 0.015), whereas Chao1 richness did not differ among groups (*p* = 0.47; Figure 2A), indicating that camel milk primarily increased microbial evenness rather than taxonomic richness. No significant differences were observed for any alpha diversity metric at the genus level (*p* > 0.05; Figure 2C).

Principal coordinates analysis based on Bray–Curtis distance revealed three distinct clusters at the genus level (PERMANOVA, R^2^ = 0.38, *p* = 0.001, Figure 2B), with model + CM positioned between control and model and closer to control (Figure 2D).

A heatmap of the top 20 most abundant genera showed that Duncaniella was more abundant in model than in control and model + CM, whereas Ligilactobacillus and Muribaculum were more abundant in model + CM than in model (Figure 2E).

LEfSe analysis (LDA score > 3.5) identified *Candidatus Arthromitus sp. SFB-mouse*, *Lactococcus lactis*, and *Klebsiella pneumoniae* as enriched in model + CM, whereas *Muribaculum intestinale* and *Sphingomonas paucimobilis* were enriched in model (Figure 2F). Differential abundance testing (model + CM vs. model; |log2FC| > 1, FDR-adjusted *p* < 0.05) further confirmed significant upregulation of *Lactococcus lactis* and *Bacteroides nordii*, as well as downregulation of *Muribaculum intestinale* (Figure 2G).

To highlight taxa potentially associated with the intervention, we selected four representative species from the above results and displayed their relative abundances across all three groups: *Candidatus Arthromitus sp. SFB-mouse* and *Lactococcus lactis* were more abundant in model + CM than in control and model; *Helicobacter hepaticus* and *Helicobacter typhlonius* were significantly more abundant in the model group than in the control or model + CM group (FDR-adjusted *p* < 0.01 for both), indicating that camel milk suppressed these pro-inflammatory pathobionts (Figure 2H).

### 3.3. Differential Analysis of Metabolites in Small Intestinal Contents

**Figure 3:**
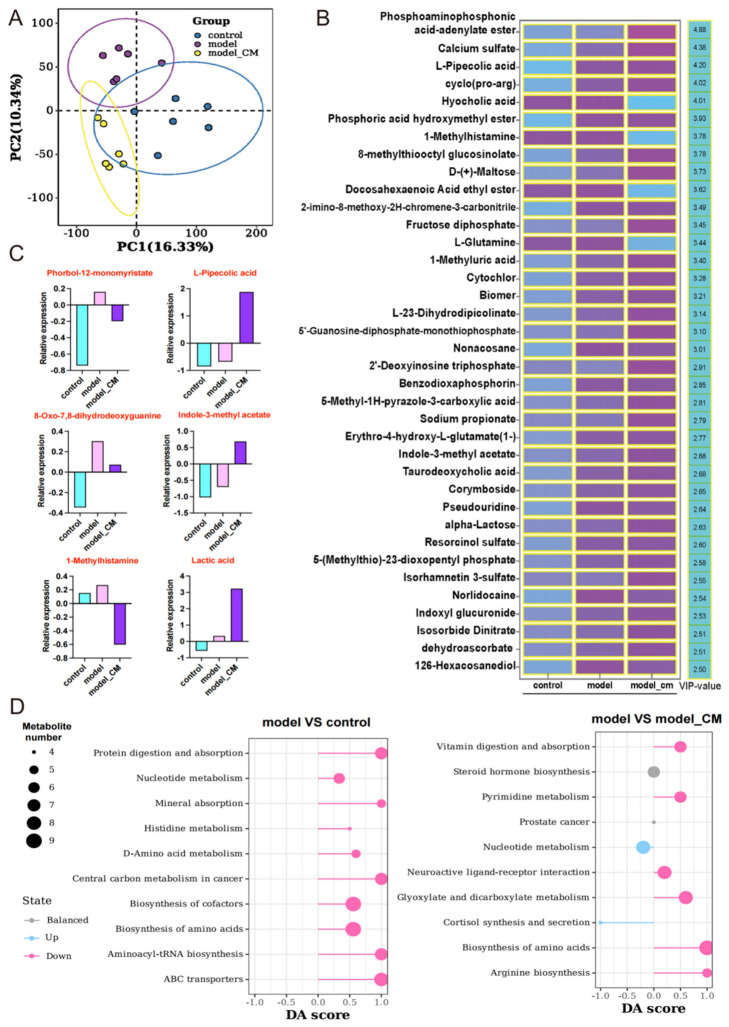
Metabolomic profiling of intestinal contents (**A**) Principal component analysis (PCA) of samples from different treatment groups; (**B**) Heatmap of differential metabolites (VIP ≥ 2.5, *p* < 0.05). Colors represent Z-score normalized relative abundance: red indicates higher abundance and blue indicates lower abundance across the three groups; (**C**) Bar plots showing relative abundance of key differential metabolites across groups; (**D**) Pathway enrichment analysis based on DA scores. The left panel shows results from the model vs. controlcomparison, and the right panel shows results from the model vs. model + CM comparison. model _CM = model + CMH, 1500 mg/kg group.

Principal component analysis (PCA) of the gut luminal metabolome revealed distinct clustering among the control, model, and model + CM groups along PC1 (16.33% variance explained) and PC2 (10.34%) (Figure 3A), with statistically significant separation between control and model groups along PC1 (*p* < 0.05), while model + CM samples occupied an intermediate position.

A total of 58 metabolites were identified as differentially abundant across groups (VIP ≥ 2.5, *p* < 0.05) (Figure 3B). Among these, compared with the control group, the model group exhibited significantly elevated levels of phorbol 12-monomyristate, 8-oxo-7,8-dihydrodeoxyadenosine, 1-methylhistamine, and indole-3-methyl acetate (*p* < 0.05). Administration of camel milk (1500 mg/kg/day) resulted in significantly reduced concentrations of phorbol 12-monomyristate, 8-oxo-7,8-dihydrodeoxyuridine, and 1-methylhistamine, while concentrations of L-pipecolic acid, lactic acid, and indole-3-methyl acetate were significantly elevated (*p* < 0.05, Figure 3C).

Pathway enrichment analysis (Figure 3D) showed that, relative to control, the model group displayed significant downregulation of ABC transporters (DA = −0.99), aminoacyl-tRNA biosynthesis (DA = −0.87), and protein digestion and absorption (DA = −0.92), alongside upregulation of central carbon metabolism in cancer (DA = 0.98) and histidine metabolism (DA = 0.85); in contrast, comparison of model + CM with model revealed downregulation of arginine biosynthesis (DA = −0.95) and amino acid biosynthesis (DA = −0.90), and upregulation of neuroactive ligand -receptor interaction (DA = 0.92) and pyrimidine metabolism (DA = 0.87).

### 3.4. Small Intestinal Microbiota-Metabolite-Intestinal Parameter Correlations

**Figure 4:**
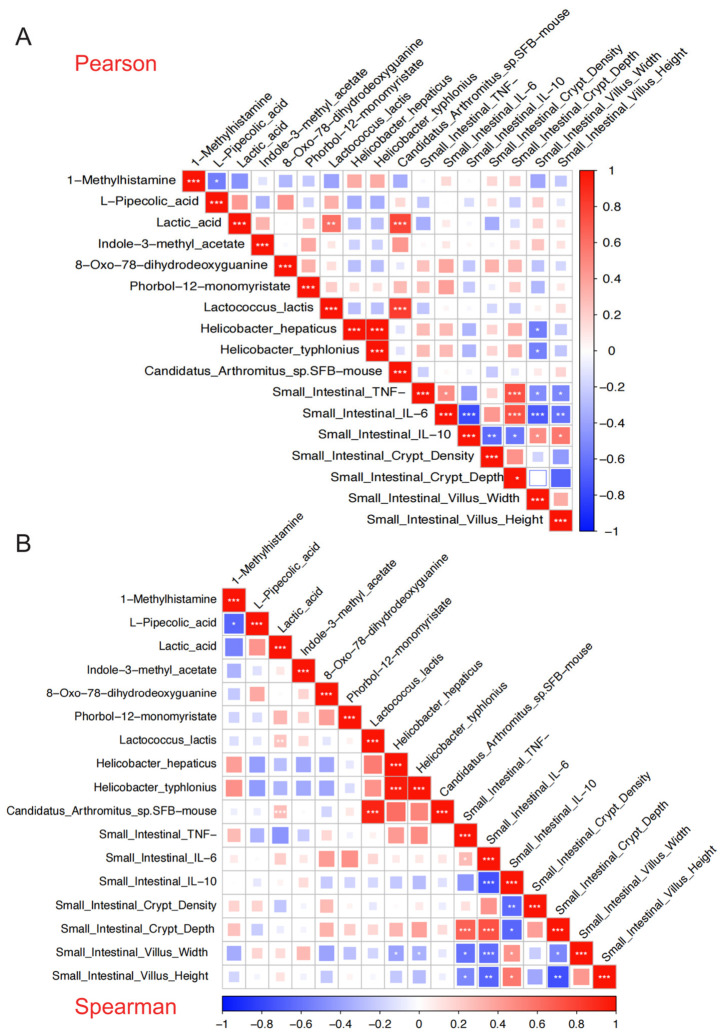
Correlation analysis between key bacteria, metabolites, and intestinal parameters. Pearson (**A**) and Spearman (**B**) correlation coefficients are shown. All reported correlations are significant after Benjamini–Hochberg FDR correction (q < 0.05). Significant correlations (*p* < 0.05) are marked with asterisks (* *p* < 0.05, ** *p* < 0.01, *** *p* < 0.001).

Pearson and Spearman correlation analyses demonstrated that Lactococcus lactis exhibited strong positive correlations with small intestinal IL-10 (r > 0.5, *p* < 0.001, Figure 4), while Helicobacter hepaticus and Helicobacter typhlonius showed strong negative correlations with small intestinal IL-10 (r < −0.5, *p* < 0.05). Lactic acid was positively correlated with small intestinal villus height (r > 0.4, *p* < 0.05) and negatively correlated with small intestinal crypt depth (r < −0.4, *p* < 0.05). Additionally, 1-Methylhistamine showed a positive correlation with small Intestinal TNF-α (r > 0.4, *p* < 0.05), and Indole-3-methyl acetate demonstrated a negative correlation with small intestinal crypt density (r < −0.4, *p* < 0.05). These findings suggest that camel milk supplementation modulates the gut microbiome and metabolite profiles, which in turn influence intestinal structural and inflammatory parameters in chronic fatigue mice, indicating a potential mechanism for the beneficial effects of camel milk on gut health in chronic fatigue conditions.

### 3.5. Small Intestinal Barrier Dysfunction and Inflammatory Markers

**Figure 5:**
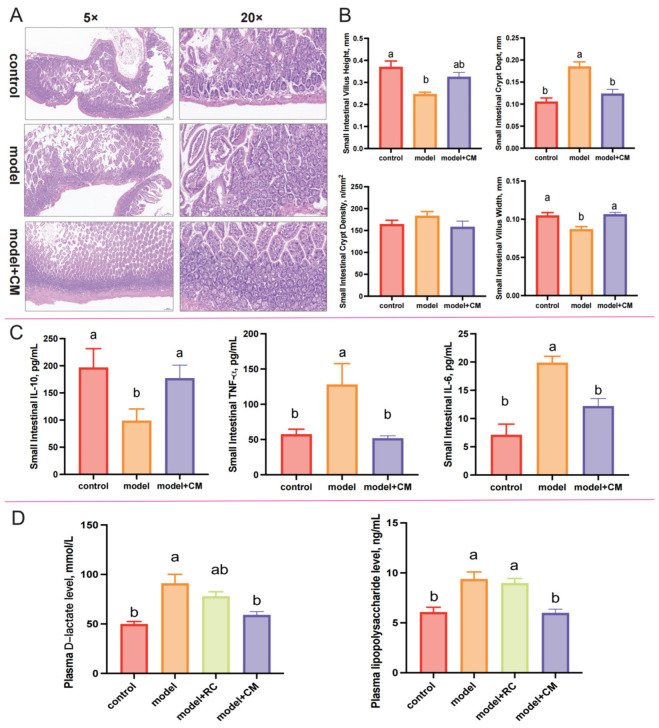
Small intestinal inflammatory markers and histopathological morphology. (**A**) Representative images of H&E-stained from control, model, and model + CM groups. Scale bars represent 200 μm at 5× magnification and 50 μm at 20× magnification. (**B**) Quantitative analysis of inflammatory indices. (**C**) ELISA-based quantification of pro- and anti-inflammatory cytokines in small intestinal tissue. (**D**) Plasma markers of intestinal integrity. The data were expressed as mean ± SEM. a, b represent a significant difference, *p* < 0.05. model + CM = 1500 mg/kg group.

H&E staining revealed that, compared to the control group, the model group exhibited shortened villi, deepened crypts, and reduced villus packing density (Figure 5A). In the model + CM group, villus length was increased and crypt depth was decreased relative to the model group, with overall tissue morphology appearing intermediate between the control and model groups (Figure 5A).

Morphometric analysis further quantified these observations. Regarding villus height, values in the model group were significantly lower than those in the control group (*p* < 0.05); the model + CM group showed significantly greater villus height compared to the model group (*p* < 0.05), although values remained lower than those in the control group (Figure 5B). For crypt depth, values in the model group were significantly higher than in the control group (*p* < 0.05); the model + CM group exhibited significantly shallower crypts compared to the model group (*p* < 0.05) (Figure 5B). No statistically significant differences in crypt density were observed among the three groups (*p* > 0.05) (Figure 5B). Crypt density did not differ significantly among groups, suggesting that camel milk primarily affects villus height and crypt depth rather than crypt number. This may indicate improved epithelial maturation without altering crypt proliferative capacity. Regarding villus width, values in the model group were significantly narrower than in the control group (*p* < 0.05), whereas the model + CM group displayed significantly wider villi compared to the model group (*p* < 0.05) (Figure 5B).

ELISA results indicated that concentrations of the anti-inflammatory cytokine IL-10 in the small intestine were significantly lower in the model group compared to the control group (*p* < 0.05) (Figure 5C). IL-10 levels in the model + CM group were significantly higher than in the model group (*p* < 0.05) and showed no significant difference compared to the control group.

For pro-inflammatory cytokines, concentrations of both TNF-α and IL-6 in the small intestinal tissue were significantly elevated in the model group compared to the control group (*p* < 0.05) (Figure 5C). Following camel milk intervention, levels of TNF-α and IL-6 were significantly reduced compared to the model group (*p* < 0.05), returning to levels that were not statistically different from the control group.

Plasma D-lactate, a sensitive indicator of enterocyte damage and paracellular permeability, was significantly elevated in the model group (*p* < 0.05 vs. control), whereas CM supplementation markedly attenuated this increase (*p* < 0.05 vs. model; Figure 5D). Similarly, plasma lipopolysaccharide levels, reflecting bacterial translocation secondary to tight junction impairment, were significantly higher in the model group compared to the control group (*p* < 0.05).

### 3.6. Plasma and Brain Inflammatory Markers

**Figure 6:**
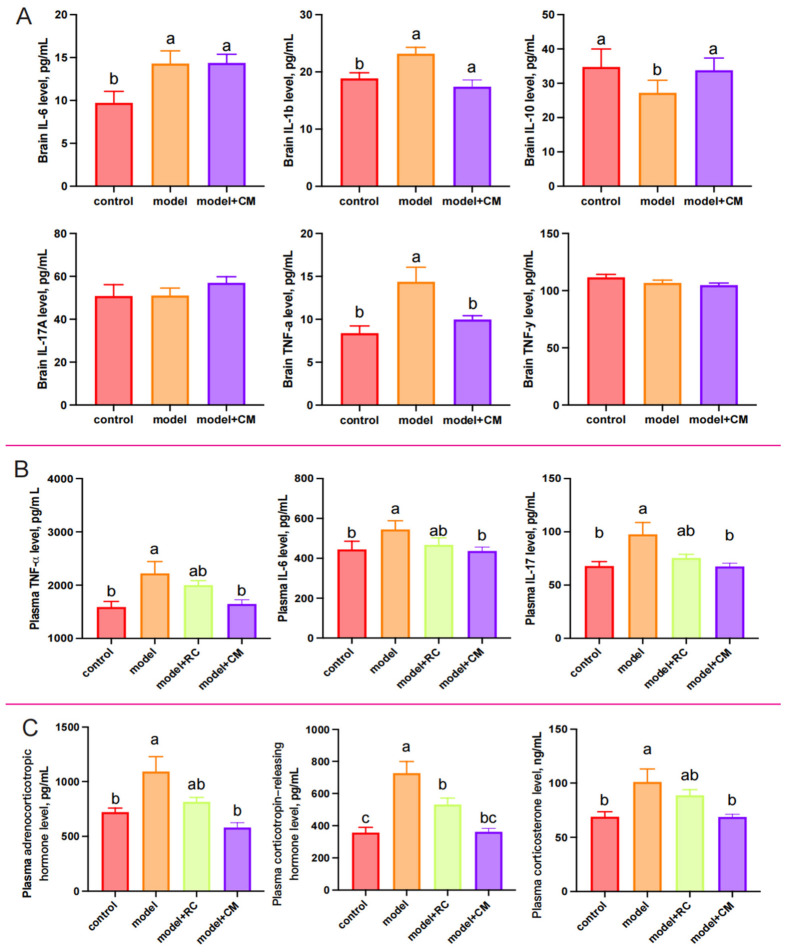
Brain and plasma inflammatory markers and HPA axis hormone. (**A**) Levels of pro-inflammatory cytokines (IL-6, IL-1β, IL-17A, TNF-α) and anti-inflammatory cytokine (IL-10) in the brain. (**B**) Levels of pro-inflammatory cytokines (TNF-α, IL-6, IL-17) in the plasma. (**C**) Levels of HPA axis hormones, including adrenocorticotropic hormone (ACTH), corticotropin-releasing hormone (CRH), and corticosterone. Data are shown as mean ± SEM. a, b represents a significant difference, *p* < 0.05. model + CM = 1500 mg/kg group.

In brain tissue (Figure 6A), compared with the control group, the model group exhibited significantly elevated levels of IL-6, TNF-α, and IL-10 (IL-6: 14.1 ± 0.81 vs. 9.5 ± 0.62 pg/mL, *p* < 0.05; TNF-α: 18.2 ± 1.11 vs. 15.1 ± 0.75 pg/mL, *p* < 0.05; IL-10: 16.3 ± 0.91 vs. 12.1 ± 0.73 pg/mL, *p* < 0.05). Compared with the CFS model group, no significant differences in brain tissue levels of IL-6, TNF-α, or IL-10 were observed in the model + CM group (*p* > 0.05). No significant differences in brain tissue IL-17A levels were detected among any of the experimental groups.

In plasma (Figure 6B), compared with the control group, the CFS model group exhibited significantly elevated levels of the pro-inflammatory cytokines TNF-α, IL-6, and IL-17 (*p* < 0.05). Compared with the model group, the CM group showed significantly reduced plasma levels of TNF-α and IL-17 (*p* < 0.05), which were comparable to those observed in the control group; no significant difference in plasma IL-6 levels was detected between the CM group and the model group. HPA axis hyperactivity in CFS mice was evidenced by elevated plasma ACTH (*p* < 0.05), CRH (*p* < 0.05), and corticosterone (*p* < 0.05) versus Control (Figure 6C). CM intervention fully normalized ACTH, CRH, and corticosterone (*p* > 0.05 vs. control; *p* < 0.05 vs. model).

## 4. Discussion

The present findings establish that camel milk intervention effectively mitigates CFS-like manifestations in a murine model, with mechanistic actions linked to remodeling of the small intestinal microbiota and the microbiota–gut–brain axis. Camel milk intervention improved behavioral performance (increased swimming time, reduced immobility, and enhanced locomotor activity), normalized peripheral fatigue markers (decreased plasma lactate, LDH, and urea), and reshaped the gut microbial structure by enriching beneficial Lactococcus lactis while suppressing pathogenic Helicobacter species. These microbial shifts were accompanied by restored intestinal barrier integrity (increased villus height, decreased crypt depth, and reduced plasma D-lactate and LPS), attenuated local intestinal inflammation (decreased TNF-α and IL-6; increased IL-10), and reduced systemic inflammation (decreased plasma TNF-α, IL-6, and IL-17). Moreover, camel milk normalized HPA axis activity, as reflected by the regulation of CRH, ACTH, and corticosterone levels. Notably, while these anti-inflammatory effects were evident in the periphery, no significant changes in brain tissue levels of IL-6, TNF-α, or IL-10 were observed following camel milk treatment. This suggests that the primary site of action for camel milk’s anti-inflammatory properties may be peripheral rather than central, or that the blood–brain barrier limited its direct impact on neuroinflammation in this model. Collectively, our findings support camel milk as a dietary intervention that targets the gut to break the cycle of inflammation and HPA axis dysregulation in CFS.

A notable consideration is that camel milk supplementation significantly increased food intake and body weight in treated mice compared to the model group. This general nutritional effect likely contributed to the observed improvements in swimming performance and metabolic markers (reduced plasma lactate, LDH, and urea), as increased caloric intake and improved nutritional status can independently enhance physical endurance and metabolic recovery. The present study design, which did not include an isocaloric control group (e.g., mice supplemented with an equal caloric load of non-bioactive protein or a standard nutritional supplement), cannot fully differentiate between camel-milk-specific bioactive effects and general nutritional support. We acknowledge that a portion of the observed benefits may be attributed to improved nutritional status rather than specific microbiota-modulating or anti-inflammatory properties of camel milk. Future studies should incorporate isocaloric control groups receiving equivalent caloric loads from other milk sources (e.g., bovine milk) or macronutrient-matched formulations to isolate the unique bioactive contributions of camel milk. While the RC group was included primarily as a positive control for behavioral validation and was not subjected to multi-omics analyses due to resource constraints, its comparable efficacy to camel milk in improving CFS-like behaviors further supports the therapeutic relevance of our intervention studies and confirms the robustness of the CFS model employed.

This study employed a combined stress model involving chronic restraint and forced swimming to simulate chronic fatigue syndrome in a mouse model. This multifactorial approach incorporates both physical exhaustion and psychological stress [38]. Behavioral validation confirmed successful model establishment. Compared to controls, model mice exhibited significantly reduced swimming duration in the forced swimming test with weights, indicating diminished endurance consistent with CFS physical exhaustion [39]; prolonged immobility in the tail suspension test, reflecting the motivational deficits commonly observed in CFS patients; and reduced locomotor activity with diminished exploration of the central area in the open field test, suggesting anxiety-like symptoms accompanying clinical CFS manifestations [40]. These Behavioral deficits were accompanied by significant metabolic disturbances, further substantiating the physiological relevance of this model. Model mice exhibited markedly elevated plasma lactate levels, reflecting enhanced anaerobic glycolysis and muscle lactate accumulation under stress; increased lactate dehydrogenase activity, a marker of muscle membrane damage and cellular injury; and elevated urea levels, suggesting heightened protein catabolism and metabolic stress during chronic stress [41,42]. Notably, moderate-dose (1000 mg/kg) and high-dose (1500 mg/kg) camel milk interventions significantly reversed behavioral deficits and metabolic abnormalities in a dose-dependent manner, with the high-dose group exhibiting the most pronounced effects. These findings confirm camel milk’s efficacy in alleviating CFS-like symptoms at the phenotypic level, establishing a robust foundation for subsequent mechanistic investigations. These benefits may stem from camel milk’s rich bioactive constituents—including immunoglobulins, lactoferrin, and essential nutrients, which possess established antioxidant and immunomodulatory properties capable of mitigating stress-induced oxidative damage and systemic inflammation [43,44].

Importantly, because metagenomic and metabolomic analyses were performed only in the high-dose group, our findings do not establish a dose-dependent relationship for the observed microbiota or metabolite changes. The dose-dependent improvements in behavioral performance (with the high dose producing the greatest effects) should be interpreted as reflecting aggregate biological outcomes that may involve multiple mechanisms beyond microbiota modulation, including nutritional support, direct anti-inflammatory effects, and other pathways not captured by our single-dose multi-omics analysis. Metagenomic analysis in this study revealed that camel milk intervention profoundly reshaped the small intestinal microbiota structure in CFS mice, with the most notable finding being the specific enrichment of beneficial bacteria and suppression of potentially pathogenic taxa. Camel milk significantly enriched *Lactococcus lactis*. *Lactococcus lactis*, a natural component of camel milk [45,46], exerts its probiotic effects through multiple mechanisms. It competitively excludes pathogens, produces lactic acid to lower intestinal pH, and generates antimicrobial compounds that strengthen epithelial barrier function. Conversely, camel milk significantly suppressed Helicobacter species (*H. hepaticus* and *H. typhlonius*). *H. hepaticus* is a well-characterised pro-inflammatory bacterium known to produce virulence factors such as cytolethal distending toxin (CdtB) [47] and to evade immune surveillance by interfering with TLR4/TLR5 signalling [48]. Infection with Helicobacter triggers Th1/Th17-mediated inflammation and disrupts intestinal barrier integrity [49]. The reduction in Helicobacter abundance by camel milk likely results from competitive exclusion by enriched probiotics, production of bacteriocins or hydrogen peroxide. Beyond these specific taxa, camel milk intervention restored overall community ecology, alpha diversity (Shannon index) was significantly increased, indicating greater species richness and evenness, while beta diversity analysis showed that the microbial structure of camel milk-treated mice clustered closer to healthy controls and away from CFS model mice. These findings precisely validate our hypothesis that camel milk alleviates CFS-like symptoms through a dual mechanism of enriching beneficial immunomodulatory bacteria while suppressing pro-inflammatory pathogens, thereby re-establishing a healthy gut microbial ecosystem.

Camel milk-induced changes in the microbial community are closely associated with profound alterations in the small intestinal metabolome, establishing a functional link between bacterial enrichment and improved gut health. Metagenomic analysis revealed significant enrichment of Lactobacillus species, directly driving metabolic remodelling within the intestinal lumen. Targeted metabolomics indicated markedly elevated lactate levels in camel milk-treated mice, reflecting the metabolic activity of enriched Lactobacillus and other lactate-producing bacteria [50]. Correlation analyses further revealed a significant positive correlation between lactate and villus height, and a significant negative correlation with crypt depth, suggesting lactate and other SCFAs may promote epithelial cell regeneration and maturation. These metabolite-microbiome interactions collectively reconfigured a microenvironment conducive to nutrient absorption and immune homeostasis. Crucially, this metabolically remodelled niche restored the physical barrier function of the gut, thereby blocking the ‘leaky gut’ pathway central to the pathogenesis of chronic fatigue syndrome. Tissue morphometric analysis confirmed that camel milk intervention reversed chronic fatigue syndrome-induced intestinal pathology, markedly increasing villus height and reducing crypt depth—morphological indicators reflecting enhanced epithelial cell renewal and regenerative capacity. At the molecular level, these structural improvements were accompanied by functional evidence of barrier repair. Plasma D-lactic acid (a sensitive marker of intestinal cell injury and paracellular permeability) and LPS (reflecting bacterial translocation due to compromised tight junctions) were significantly elevated in model mice but markedly decreased following camel milk intervention. The reduction in plasma LPS was particularly significant, indicating effective suppression of endotoxin translocation, a key initiating event in CFS-associated systemic inflammation [51,52]. Mechanistically, the observed barrier enhancement aligns with the known probiotic functions of lactic acid bacteria: Lactobacillus species and related strains can upregulate tight junction proteins and enhance epithelial integrity via metabolite-mediated signalling pathways [53]. Furthermore, studies indicate that lactic acid-producing bacteria mitigate LPS leakage by strengthening the gut–liver axis and reducing systemic endotoxin exposure [54]. Collectively, these findings delineate a coherent mechanistic cascade: camel milk, by enriching lactic acid-producing microbiota, drives metabolic remodelling that synergistically restores intestinal barrier integrity, thereby effectively disrupting the vicious cycle of LPS translocation and systemic inflammation characteristic of CFS. This ‘microbiota-metabolite-barrier’ axis provides a mechanistic framework for understanding camel milk’s therapeutic effects and supports its potential as a functional food intervention for gut health.

The resolution of intestinal inflammation represents the critical nexus linking camel milk-induced microbial remodelling to systemic benefits. Camel milk intervention significantly attenuated local inflammation in the small intestine, as evidenced by reduced pro-inflammatory cytokines (TNF-α, IL-6) and restored anti-inflammatory IL-10 levels—a bidirectional modulation of the “inflammatory cytokine network” consistent with previous reports on camel milk’s immunoregulatory properties [26]. This anti-inflammatory effect was mechanistically coupled with enhanced intestinal barrier function. While our study did not directly measure the expression of tight junction proteins (claudin-1, occludin, ZO-1), the significantly reduced plasma D-lactate and LPS levels, together with histomorphological improvements (increased villus height, reduced crypt depth), provide functional evidence of enhanced intestinal barrier integrity. These indirect markers are well-established indicators of paracellular permeability and epithelial integrity in murine models. Based on these functional data, we infer that camel milk likely enhances tight junction protein expression, consistent with previous reports in other models. However, we explicitly acknowledge the absence of direct molecular evidence; future studies should incorporate immunohistochemistry or Western blotting to confirm the effects of camel milk on claudin-1, occludin, and ZO-1 expression [55]. The “microbiota-inflammation” axis played a central role in this process. Camel milk enriched beneficial bacteria (e.g., *Lactococcus lactis*, *Ruminococcaceae*) that positively correlated with intestinal IL-10, likely through induction of regulatory T cells (Treg) or production of SCFAs [56]. Concurrently, it suppressed pro-inflammatory Helicobacter species that negatively correlated with IL-10, thereby reducing the local inflammatory burden. By restoring intestinal barrier integrity and resolving local inflammation, camel milk effectively blocked the pathway through which gut-derived inflammatory signals propagate to distal organs. This was reflected in significantly reduced plasma pro-inflammatory cytokines (TNF-α, IL-17), demonstrating successful containment of the “inflammatory storm” and prevention of systemic dissemination [57,58]. Thus, the intestine serves as the primary site of immune modulation and homeostasis, and camel milk, by restoring gut homeostasis, establishes a foundation for systemic immunological balance.

The restoration of hypothalamic–pituitary–adrenal (HPA) axis function represents the neuroendocrine culmination of camel milk’s multi-level effects, directly translating into behavioral improvement. CFS model mice exhibited characteristic HPA axis hyperactivity, with significantly elevated plasma ACTH, CRH, and corticosterone—hallmarks of stress-induced neuroendocrine dysregulation frequently observed in CFS patients [59]. Camel milk intervention completely normalized all three hormones, restoring them to control levels. This normalisation is particularly significant, as HPA axis dysfunction directly affects energy metabolism, stress resilience, and behavioral performance. The recovery of HPA axis function was closely associated with reduced anxiety-like behaviour: camel milk-treated mice showed increased centre zone exploration in the open field test, indicating diminished anxiety—a behavioral improvement mechanistically linked to normalized corticosterone levels [60]. While camel milk did not significantly alter brain inflammatory cytokine levels, the restoration of HPA axis homeostasis likely resulted from reduced peripheral inflammatory signalling reaching the brain, possibly via the vagus nerve or circulating prostaglandins, rather than direct central anti-inflammatory effects. SCFAs, elevated following camel milk intervention, have been shown to directly influence HPA axis function and stress responses through GPR43-mediated pathways or by modulating blood–brain barrier permeability [61]. While camel milk did not significantly alter brain inflammatory cytokine levels, the restoration of HPA axis homeostasis likely resulted from reduced peripheral inflammatory signalling reaching the brain, possibly via the vagus nerve or circulating prostaglandins, rather than direct central anti-inflammatory effects.

It is important to acknowledge that the present study utilized lyophilized (freeze-dried) camel milk rather than raw or pasteurized products. Lyophilization was chosen for experimental standardization, extended shelf-life, and precise dosage control. However, processing methods differentially affect the structural integrity and bioactivity of camel milk immunoproteins. Freeze-drying preserves the native conformation of lactoferrin and immunoglobulins more effectively than thermal processing, maintaining their LPS-neutralizing capacity and receptor-binding activities. In contrast, pasteurization induces partial protein denaturation and aggregation, which may reduce the bioavailability of these bioactive components. Thus, the beneficial effects observed in our study should be interpreted in the context of lyophilized camel milk, and the translational potential to pasteurized commercial products---which are more commonly available for human consumption---requires direct validation. Nevertheless, the lyophilized preparation employed here provides a standardized, well-characterized experimental matrix that allows reproducible investigation of the mechanistic pathways underlying camel milk’s gut-protective effects. Several limitations of this study should be acknowledged. First, while our data demonstrate strong associations between camel milk-induced microbial changes and phenotypic improvements, the evidence remains primarily correlational; fecal microbiota transplantation experiments are essential to establish whether the altered microbiota is causally sufficient to confer the anti-fatigue phenotype. Second, the neural pathways mediating gut–brain communication—particularly vagal afferent signaling—were not directly investigated, leaving the relative contributions of humoral versus neural routes unresolved. Third, the exclusive use of male mice limits generalizability, as CFS exhibits marked sex bias with higher prevalence in women, and potential sex differences in treatment response remain unexplored. Fourth, the exclusive use of male mice represents a significant limitation, as CFS exhibits a marked sex bias with higher prevalence in women, and potential sex differences in treatment response remain completely unexplored. This is particularly critical given that sex hormones influence both immune function and gut microbiota composition, and the mechanisms identified here may not fully translate to female subjects. Future studies should include both male and female mice to evaluate sex-dependent responses to camel milk intervention, and the present findings should be interpreted as specifically applicable to male mice until such data are available. We explicitly caution against generalization of these results to female populations. Fifth, while the chronic restraint plus forced swimming paradigm recapitulates key features of stress-induced fatigue, it may not fully capture the etiological heterogeneity of human CFS, which can arise from infectious, immune, or metabolic triggers; validation in additional CFS models would strengthen the generalizability of our conclusions. Sixth, the present study did not directly assess the expression of key intestinal tight junction proteins (e.g., claudin-1, occludin, ZO-1). Although elevated plasma D-lactate and LPS levels provided indirect evidence of increased intestinal permeability, and H&E staining revealed morphological changes, molecular-level confirmation is lacking; future studies should incorporate immunohistochemistry or Western blotting to validate the effects of camel milk on tight junction protein expression.

Seventh, our investigation only measured endpoint outcomes after 56 days of intervention; the long-term safety of camel milk supplementation and the persistence of its beneficial effects after discontinuation remain unknown. Eighth, while the high dose (1500 mg/kg/day) showed the most pronounced efficacy, a full dose–response curve with nonlinear modeling was not performed, and the optimal therapeutic dose requires further refinement. Finally, the findings are derived from a mouse model; clinical efficacy and appropriate dosage of camel milk in CFS patients need to be validated through well-designed randomized controlled trials.

## 5. Conclusions

In summary, our findings indicate that lyophilized camel milk administration at 1500 mg/kg/day correlates with amelioration of CFS-like symptoms in mice, with observed effects associated with microbiota–gut–brain axis modulation. Oral administration of camel milk was associated with reshaping of the small intestinal microbiota, specifically enriching *Lactococcus lactis* while suppressing pathobionts such as *Helicobacter hepaticus* and *Helicobacter typhlonius*, which correlated with a favorable metabolic shift characterized by elevated luminal lactic acid and reduced oxidative and inflammatory mediators. These microbial and metabolic changes were closely associated with structural and functional restoration of the intestinal barrier, as evidenced by increased villus height, reduced crypt depth, downregulation of local pro-inflammatory cytokines (TNF-α, IL-6), and upregulation of anti-inflammatory IL-10. Critically, this mucosal healing was associated with attenuated bacterial translocation, as confirmed by decreased plasma D-lactate and lipopolysaccharide, thereby correlating with reduced systemic inflammation (reduced TNF-α and IL-17) and normalization of HPA axis hyperactivity. Our findings establish associations between camel milk intervention and microbiota-gut–brain axis modulation, supporting its potential as a microbiota-targeted functional food. However, we emphasize that these findings are correlational; fecal microbiota transplantation and other causal mechanistic studies are needed to definitively establish whether the altered microbiota is causally responsible for the observed anti-fatigue effects.

While our findings demonstrate the therapeutic potential of camel milk for CFS-like symptoms, the observed enrichment of *Klebsiella pneumoniae* in the model + CM group warrants careful consideration. *K. pneumoniae* is an opportunistic pathogen that can cause severe infections, particularly in immunocompromised individuals. Although no signs of infection were observed in our healthy mice, the long-term safety of camel milk supplementation in immunocompromised populations, including those with primary immunodeficiency, undergoing chemotherapy, or with chronic conditions such as diabetes or liver cirrhosis, cannot be assumed. Future studies should incorporate comprehensive safety assessments, including monitoring for *K. pneumoniae* translocation, systemic dissemination, and virulence gene expression, particularly when considering camel milk as a therapeutic intervention for vulnerable patient populations. Clinicians should exercise caution and conduct individualized risk-benefit assessments before recommending camel milk to immunocompromised CFS patients. While our findings in male BALB/c mice are promising, they should be interpreted with caution regarding translational application to human CFS patients, particularly given the predominantly female prevalence of the condition and the unknown sex-specific responses to camel milk intervention.

## Data Availability

Data will be made available on request. The raw metagenomic sequencing reads have been submitted to the NCBI Sequence Read Archive (SRA) under BioProject PRJNA1433199 (https://www.ncbi.nlm.nih.gov/bioproject/PRJNA1433199, accessed on 6 May 2026). The raw Metabolomics datasets have been deposited in the NGDC OMIX repository under the accession number OMIX016357 (https://ngdc.cncb.ac.cn/omix/release/OMIX016357 (accessed on 19 April 2026)).

## Supplementary Materials

The following supporting information can be downloaded at: https://www.mdpi.com/article/10.3390/foods15142451/s1, Table S1. Metagenomic sequencing depth, data quality metrics, and assembly outcomes for all samples.
