# Supplementary material for: Camel Milk Alleviates Chronic Fatigue Syndrome-like Symptoms in Mice by Modulating the Small Intestinal Microbiota and Inflammation
Source: Foods. 2026 Jul 10;15(14):2451. doi: 10.3390/foods15142451 (PMC13409245; doi:10.3390/foods15142451)
Supplement: Supplementary file 1 [file foods-15-02451-s001.zip › foods-4384378-supplementary.pdf]

Supplementary Table S1. Metagenomic sequencing depth, data quality metrics, and assembly outcomes for all samples.

Sample	Raw Reads	Raw Bases	Clean Reads	Clean Bases	Clean Data Rate	Q20	Q30	GC	Contig Number	Assembly Length	N50	N90	Max	Min	Average Size
model1	230880000	23088000000	200559316	20055931600	86.87	98.1	92.46	44.13	187091	190415884	1358	434	167425	300	1017
model2	225040000	22504000000	200417580	20041758000	89.06	98.69	94.04	42.08	18214	14495523	871	364	186500	300	795
model3	238480000	23848000000	200430906	20043090600	84.05	98.01	92.29	41.99	65437	58747673	1058	414	134795	300	897
model4	231360000	23136000000	200360122	20036012200	86.6	98.11	92.57	43.06	259436	277841583	1524	438	301264	300	1070
control1	223120000	22312000000	200303260	20030326000	89.77	98.51	93.83	42.7	74520	93413299	2476	452	301286	300	1253
model5	233760000	23376000000	200454728	20045472800	85.75	98.19	92.57	43.44	28506	31138229	1485	452	301263	300	1092
control2	236880000	23688000000	200252456	20025245600	84.54	98.23	92.87	43.28	124377	155699668	2211	466	322574	300	1251
control3	219120000	21912000000	200223926	20022392600	91.38	97.79	91.41	42.31	27391	25871808	1062	431	301264	300	944
control4	219040000	21904000000	200375676	20037567600	91.48	98.04	92.2	44.49	176968	243562646	2408	508	325242	300	1376
control5	265840000	26584000000	200279656	20027965600	75.34	98.41	93.31	42.69	347463	184021504	526	346	193206	300	529
model+CM1	232800000	23280000000	200325028	20032502800	86.05	98.22	92.7	40.74	4459	2855970	651	370	39037	300	640
model+CM2	226080000	22608000000	200429248	20042924800	88.65	97.82	91.56	41.64	3571	2101043	586	356	11617	300	588
model+CM3	237120000	23712000000	200414676	20041467600	84.52	98.03	92.04	42.28	8168	6286610	867	385	37303	300	769
model+CM4	262800000	26280000000	200259554	20025955400	76.2	98.45	93.95	41.82	306640	350776061	1826	443	279144	300	1143
model+CM5	214560000	21456000000	200346660	20034666000	93.38	98.3	93.54	44.92	6760	4565518	714	361	25219	300	675
